# Prophylactic salpingo-oophorectomy & surgical menopause for inherited risks of cancer: the need to identify biomarkers to assess the theoretical risk of premature coronary artery disease

**DOI:** 10.1186/s40695-018-0037-y

**Published:** 2018-04-27

**Authors:** Zarah Batulan, Nadia Maarouf, Vipul Shrivastava, Edward O’Brien

**Affiliations:** 1 Department of Cardiac Sciences, Libin Cardiovascular Institute of Alberta, University of Calgary, Health Research Innovation Centre, GB42, 3280 Hospital Dr NW, Calgary, AB T2N 4Z6 Canada; 2 Department of Cardiac Sciences, Libin Cardiovascular Institute of Alberta, Health Research Innovation Centre, Room GAA16, 3280 Hospital Drive NW, Calgary, AB T2N 4Z6 Canada

**Keywords:** Atherosclerosis, Cardiovascular disease, Cardiovascular risk factors, Coronary artery disease, Estrogen, Heat shock protein 27, HSP27, Inflammatory cytokines, Menopause, PCSK9, Prophylactic salpingo-oophorectomy, Retinal vessel analysis, Surgical menopause

## Abstract

**Background:**

Some women with genetic risk of breast and/or ovarian cancer (e.g., BRCA1/2) opt to undergo prophylactic salpingo-oophorectomy (PSO, or surgical removal of the ovaries & fallopian tubes) in order to reduce their risk of cancer. As a consequence, these women experience “surgical menopause” – accompanied by more severe climacteric symptoms that occur in a much shorter time frame. While the risk of coronary artery disease (CAD) rises with menopause, little is known about how the sudden loss of ovarian function from PSO alters the whole-body physiology, and whether it predisposes women to premature CAD.

**Methods/Design:**

To manage CAD risk there is a prerequisite for reliable biomarkers that can help guide risk assessment and therapeutic interventions. To address these needs, this prospective, observational cohort study will evaluate surrogate markers reflective of CAD health in women experiencing surgical menopause after PSO. Twenty women representing each of the following groups will be enrolled over 3 years (total participants = 240): (i) pre-menopausal PSO, (ii) post-menopausal PSO, (iii) pre-menopausal women undergoing other pelvic surgery, and (iv) pre-menopausal controls (no surgery). All participants will provide blood plasma samples pre- and 1, 3, 6, & 12 months post-operatively, with serial samples collectively assessed for measurements of the study’s primary endpoints of interest. These include a hormone profile (estradiol, follicle stimulating hormone (FSH), luteinizing hormone (LH), and progesterone) and both conventional (lipid profile) and novel biomarkers (Heat Shock Protein 27 (HSP27), HSP27-antibodies (HSP27 Ab), proprotein convertase subtilisin/kexin 9 (PCSK9), inflammatory cytokines) of CAD. Another aspect of this study is the measurement and analysis of retinal vessel diameters – an emerging physiological parameter reflective of CAD risk. Finally, a patient engagement exercise will result in the drafting of patient-generated questionnaires that address the well-being and health concerns of these women as they transition through premature menopause and work with our research team to identify and discuss their health priorities.

**Discussion:**

The protocol of our planned study investigating the effects of PSO on CAD is described herein. Characterization of novel CAD markers in women experiencing surgical menopause will yield new insights into the role of the functional ovary in modulating lipid parameters and other CAD risk factors such as HSP27 and HSP27 Ab.

## Background

It is widely recognized that pre-menopausal women are relatively protected from cardiovascular disease (CVD), including the manifestations of (*i*) coronary artery diseases (CAD, e.g., angina & heart attacks), and (*ii*) stroke. However, this advantage is lost after menopause [1–3]. In fact, one-third of all deaths in post-menopausal women are due to CAD [4], with a higher risk and overall mortality in women who experience premature or early-onset menopause [5]. As more women worldwide with hereditary breast and ovarian cancer mutations consider surgical removal of their ovaries and fallopian tubes (“prophylactic salpingo-oophorectomy”, PSO) to reduce their cancer risk, it is increasingly important to highlight the potential cardiovascular risks that accompany such an intervention and the ensuing premature, “surgical” menopause. The aim of this study will thus be to investigate whether PSO leads to increased cardiovascular risk as assessed by the measurement of conventional and novel surrogate markers for CAD. The rationale and methodology is thereby presented herein as a *study protocol* manuscript.

### Menopause

Menopause is a complex physiological process that results from reduced secretion of ovarian hormones, estrogen and progesterone. Clinical manifestations of this physiological transition include vasomotor symptoms e.g. ‘hot flashes’, urogenital problems, sexual dysfunction, sleep disturbances, depression and osteoporosis [6]. Another common clinical presentation post-menopause is accelerated changes in CAD risk factors, as first reported in the Healthy Women Study (HWS) [7, 8], and later supported by various other studies, including the Study of Women Across the Nation (SWAN) [9]. These observations led to the assumption that estrogens are cardio-protective, a theory which was initially supported by retrospective observational studies in women [1, 10, 11], and later by in vivo animal studies demonstrating that estrogen exerts beneficial physiological effects on: i) the vascular endothelium; and ii) plasma lipoprotein profiles (e.g., by increasing levels of “good” cholesterol – high density lipoprotein (HDL) – while reducing insulin resistance biomarkers [12, 13]).

Evidence accumulated at that time indicated that increased CAD risk post-menopause most likely occurs because of a loss of ovarian hormones. Therefore, several subsequent clinical trials tested the therapeutic potential of post-menopausal hormone therapy (MHT). However, the cardio-protective hypothesis of MHT was refuted by several major randomized clinical trials assessing both primary and secondary CAD prevention (these included the Women’s Health Initiative (WHI) and the Heart Estrogen/Progestin Replacement Study (HERS) [14, 15]). In fact, the early termination of the WHI study occurred when the safety monitoring board concluded that the risks of using MHT to prevent / treat CAD exceeded the benefits. This trial brought into sharper focus the conclusion that MHT is associated with adverse cardiovascular outcomes and increased risk of stroke, thromboembolism and breast cancer, most likely through changes in the blood coagulation index favoring the direction of enhanced clotting and increased inflammation [16]. Consequently, there was a marked decline in MHT use worldwide. More recently, this discordance between the theoretical benefit of MHT and the clinical outcomes of the randomized clinical trials has undergone critical reappraisal and new experimental data strongly argued for a second look at MHT [17, 18]. An important fact which may help explain the incongruity between the expected athero-protective effects of MHT and the lack of cardiovascular benefits in patients is the ‘timing hypothesis’. Based on the premise that MHT affects CAD outcomes more favorably in younger, compared to older women [19], this hypothesis has drawn attention to the fact that in the WHI study the introduction of MHT was, on average, 7 years post-menopause and probably too late to generate athero-protective benefits. Moreover, there is the theoretical argument that compared to arteries free of atherosclerosis, estrogen receptor responsiveness in CAD may be diminished [20]. Thus, exogenous estrogens (i.e., MHT) may be ineffective late after the onset of menopause if CAD is already present or progressing. Unfortunately, there are currently few (if any) robust therapeutic options to lower cardiovascular risk for post-menopausal women – and to a large degree, this reflects the lack of enrollment and study of women in cardiovascular prevention trials. While this neglectful inattention to the plight of coronary artery disease in women has a history that spans decades [21], even the most important cardiovascular trials reported in 2017 have woefully low percentages of women enrolled in them (e.g., 16–26%) [22].

### “Surgical” menopause

In the first half of the twentieth century, there were already reports of European women undergoing surgical removal of the ovaries and fallopian tubes (or prophylactic salpingo-oophorectomy, PSO) to prevent the onset and/or progression of breast cancer. Hence, more than 100 years ago, female hormones, particularly estrogens, were already being implicated in tumorigenesis. Unfortunately, the negative impact of removing the source of estrogens (via surgical intervention) on a woman’s cardiovascular (and cognitive) health was not considered nor deemed important – most likely because it seemed to be the lesser of two evils (i.e., risk of cancer outweighing cardiovascular disease). Historically, community-based studies for CAD did not focus on women; however, epidemiological studies in the mid-1900s began to indicate that women have a lower tendency of developing cardiovascular disease compared to men – a situation that reverses after menopause. Indeed, concerns surfaced regarding the cardiovascular health of women who underwent PSO and the ensuing “surgical” menopause. An illustrative example is a study of Scottish women (≤ 35 yrs. old) who underwent PSO between 1934 and 1938 and then followed for 20 years post-surgery, showing higher CAD events in PSO patients compared to controls [23]. These findings were independently observed by other research groups during that time [23–29].

Advances in genetic diagnostic testing within the past decade have led to the early identification and detection of women with hereditary cancer-causing mutations [30, 31]. The popular actress and human rights advocate, Angelina Jolie, was one such woman diagnosed with a breast cancer-causing BRCA1/2 genetic mutation, who opted to undergo PSO to reduce her risk of developing cancer. Her personal health saga was widely covered by the mass media, and consequently, the number of women diagnosed with BRCA-related mutations, as well as instances of PSO intervention, rose significantly [32, 33]. Less highlighted, however, was the possible negative impact on cardiovascular health. The need to balance the cancer-sparing benefit of PSO with its theoretical risk of premature CAD is thus starting to emerge. Now is the time for women who are considering surgical removal of their ovaries and fallopian tubes (to lower their hereditary cancer risk) to be aware of the cardiac health risks they may face in the future.

### PSO as clinical intervention to reduce cancer risk

**Fig. 1:**
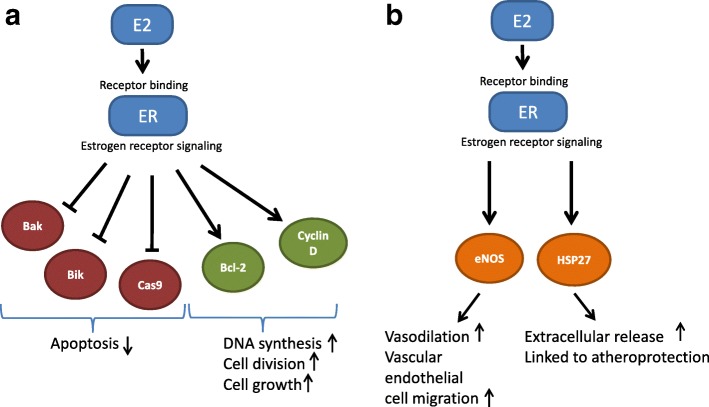
Estrogen binding to its receptors activate signaling pathways that promote cell division & proliferation in breast cancer cells (**a**), and that regulate vasodilation & HSP27 release in the cardiovascular system (**b**). E2: estrogen, ER: estrogen receptor, Cas9: caspase 9, eNOS: endothelial nitric oxide synthase, HSP27: heat shock protein 27. [34–36, 57, 68, 94]

Historically, it appears that removal of the ovaries and fallopian tubes delayed and/or reduced the severity of breast and ovarian cancers, despite the lack of a clear understanding of how precisely estrogens (and other female hormones) instigate and/or regulate cancer onset and progression. Nowadays, there is a better mechanistic understanding of how estrogen, which is essentially a proliferative factor, can promote the dysregulated growth of tumor cells (Fig. 1a) [34–36]. Women with genetic predispositions to ovarian cancer may thus opt to ablate/eliminate/remove their ovaries – their main source of estrogen [37]. As well, since there is growing concern that the fallopian tubes may also be a source of malignant cells, they are also removed. Currently, the following genes are the most commonly linked to hereditary breast & ovarian cancers:

### BRCA1/2

It is estimated that ~ 15% of women with epithelial ovarian cancer have mutations in BRCA1 or 2 [38]. Both genes were first implicated as causative factors for hereditary breast and ovarian cancers in the mid-1990s [39, 40] and have since been characterized extensively as multifunctional proteins that play key roles in DNA repair, replication, chromatin stability, and cell cycle control [41]. Given its importance in promoting genomic stability, its high disease penetrance (30–70% lifetime risk for cancer) is not surprising. To date, 61 missense mutations (70.5% in BRCA1 & 29.5% in BRCA2) have been documented [42]; however, it is not yet clear if patients have higher cancer risk depending on the type of BRCA mutation. Regardless of the actual cancer risk inherent in a particular mutation, an increasingly common clinical recommendation for BRCA1/2 gene mutation carriers is PSO, as it has been shown by several groups to lower risk of ovarian, fallopian tube, and epithelial cancer by 80%, and reduce mortality risk by 77% [37, 43–45].

### Lynch syndrome-associated genes

Women with Lynch syndrome, caused by mutations in DNA mismatch repair genes (MSH2, MLH1, MSH6, PMS2, EPCAM), usually develop colorectal cancer but may also be at high risk for ovarian and endometrial cancers [46]. These women may also be advised to undergo PSO to lower their risk of ovarian cancer [47].

### PSO, surgical menopause, and increased cardiovascular risk

Although PSO may lower cancer risk, the effect of surgical menopause leads not only to more severe menopausal symptoms [48], but also elevated risks in cardiovascular disease particularly in younger pre-menopausal women (< 45 years of age at surgery) [23, 49]. The abrupt drop of serum sex hormones (i.e., estrogen), is associated with a doubled risk of myocardial infarction, an increase in the relative risk for fatal & nonfatal coronary heart disease, stroke, as well as higher incidence of metabolic syndrome [50, 51]. There are also increased concerns regarding an accelerated risk of dementia in women who have undergone PSO at young, pre-menopausal ages [52, 53]. Post-menopausal dementia may be a form of vascular cognitive impairment, possibly attributable to inflammatory cytokines [54]. Recent epidemiological studies have associated bilateral oophorectomy to increased rates of multimorbidity (as defined by the assessment of chronic conditions associated with aging, including indices of cardiovascular and mental health) [49, 55] suggesting that PSO can accelerate the aging process.

How does the sudden drop in estrogen post-PSO worsen women’s cardiovascular risk profile? Atherosclerosis is driven by both inflammation and cholesterol accumulation, and since estrogen’s multitude of physiological effects include the attenuation of both of these processes, its abrupt loss after PSO can thus aggravate atherogenesis. Estrogen has both direct and indirect effects on the endothelium of the vasculature, acting as a vasodilator and helping to repair damaged endothelium via recruitment of new endothelial cells and its action on immune cells (Fig. 1b) [56–58]. Related to lipid regulation, estrogen is inversely correlated with serum levels of low density lipoprotein (LDL) and PCSK9 [59, 60], a recently characterized negative mediator of cholesterol metabolism.

### Surrogate markers of cardiovascular risk

#### HSP27

Over the past 15 years, our group has also focused on HSP27, a molecular chaperone protein that associates with estrogen receptor-beta. It was rather fortuitous that we made a key clinical observation that HSP27 protein expression in human coronary arteries diminishes as the stage of coronary atherosclerosis advances [61]. Interestingly, four other groups using objective (proteomic discovery) approaches, also demonstrated that expression of HSP27 diminishes with the development and progression of atherosclerosis. [62–65] Of clinical importance, we found that HSP27 is a novel cardiovascular biomarker, as elevated serum levels are associated with a lower 5-year risk of having a myocardial infarction, stroke or cardiovascular death [66]. HSP27 is more than a conventional intracellular chaperone protein as it has an extracellular signaling function that is instrumental in protecting against the development of atherosclerosis [67]. For example, we showed in atherosclerosis-prone apolipoprotein E null (ApoE^−/−^) mice that augmentation of extracellular HSP27 levels reduces both serum and plaque cholesterol content, as well as promotes the formation of plaques with histological features of enhanced plaque stability [66, 68, 69]. The mechanisms by which HSP27 reduces atherogenesis appear to be distinct from its chaperone function. From our initial studies we suggested that this atheroprotection occurred because HSP27 promoted the release of the anti-inflammatory chemokine IL-10 [68, 70] and attenuated foam cell formation by binding to and/or reducing the expression of scavenger receptor AI (SR-AI) [68, 71]. We now believe that the cholesterol lowering effect of HSP27 is due to its modulation of a key mediator of cholesterol metabolism, PCSK9, and that estrogens may play an important role in this process.

#### HSP27 Ab

Recent findings in the O’Brien Laboratory suggest that HSP27 Ab are significantly lower in coronary artery disease patients compared to controls (unpublished data). Others have shown variable HSP27 Ab levels in the context of heart disease, with elevations first noted during acute coronary events followed by a rapid drop [72] and both increases and decreases observed in cardiovascular disease patients (unpublished data) [73, 74]. The functional role of circulating HSP27 Ab is unclear and currently is the subject of much study in our laboratory.

#### PCSK9

Interest in PCSK9 arose in 2003 when a gain-of-function *PCSK9* mutation was linked with autosomal dominant hypercholesterolemia [75]. This was subsequently confirmed when loss-of-function mutations in PCSK9 were shown to lower LDL [76]. Later experiments indicated that PCSK9 interferes with clearance of LDL from the circulation by binding to LDL-receptor (LDL-R) and facilitating its degradation [77]. Higher circulating plasma levels of PCSK9 has been shown to positively correlate with plasma levels of LDL and susceptibility to CAD; individuals with a PCSK9 loss of function display reduced LDL levels and lower risk of CAD [78]. Findings from the Dallas Heart Study suggest that PCSK9 levels are higher in post- menopausal compared to pre-menopausal women [79]. Of note, our laboratory obtained promising in vitro and in vivo results supporting the role of recombinant HSP27 in reducing PCSK9 levels in both liver cells / tissue and serum (unpublished data).

#### Retinal vessel analysis

With advances in technology, relatively low cost, excellent reproducibility and radiation free testing, assessment of retinal arterioles is an attractive screening modality for assessing CAD risk [80–84]. Recently, changes in retinal microvessels were reported to correlate with cardiovascular outcomes [85]. Narrower retinal arterioles and wider retinal venules were associated with increased long-term risk of cardiovascular mortality and ischemic stroke both in male and female patients. Measurements of the central retinal arteriolar equivalent (CRAE) and the central retinal venular equivalent (CRVE) were also linked to cardiovascular disease, and in women were predictive of stroke [85]. Hence, retinal arteriolar narrowing is an early marker of systemic microvascular dysfunction with a higher sex-specific risk predictability for atherosclerotic disease in women [85]. Studies are ongoing to determine if retinal vessel parameters are static or can improve with cardiovascular risk factor modification (e.g., ClinicalTrials.gov identifiers: NCT02853747 and NCT02796976) [86]. Therefore, we are using retinal vessel analysis as a surrogate CAD endpoint as it has previously been indicated to be predictive of atherosclerotic events (i.e., small arteriole: large venule diameter ratio) [85].

This study will address the hypothesis that PSO intervention in pre-menopausal women, which causes an abrupt drop in estrogen and premature menopause, leads to physiological changes that increase cardiovascular risk. Surrogate markers for CAD (conventional and novel biomarkers, as well as measurement of an emerging physiological parameter reflective of CAD – retinal vessel analysis) will be used as tools to evaluate cardiovascular risk, with the long-term goal of potentially using these markers as predictive determinants of cardiovascular health in other at-risk populations.

## Methods / design

### Study design

This is a single-center, prospective, cross-sectional study which addresses whether women undergoing PSO develop features of cardiovascular disease as assessed by conventional (e.g., cholesterol) and novel biomarkers, including HSP27. Candidates for surgery will be introduced to the study by medical genetics counselors and gynecologists specializing in minimally invasive PSO (University of Calgary’s affiliated hospitals, Calgary, Alberta). Patients who decide to undergo surgery and are interested in participating in this study are then scheduled for a cardiac consultation with EOB (University of Calgary). To determine if the sudden drop in estrogen following PSO leads to increases in cardiovascular disease markers, the study population will involve women representing the following four different treatment groups:

1. *Pre-menopausal women undergoing PSO.* This patient group is predicted to develop the most obvious changes in cardiovascular disease markers, as previously indicated by increases in total cholesterol and LDL levels [59, 60] – this is most likely attributed to the abrupt loss in estrogen after surgical removal of functional ovaries.
2. *Post-menopausal women undergoing PSO.* Comparison with this group of women will address whether the surgical removal of ovaries alone (which are non-cycling), and not the sudden drop in estrogen per se, contributes to increases in cardiovascular disease markers.
3. *Pre-menopausal women experiencing other pelvic surgeries* that spare both ovaries and fallopian tubes (e.g., hysterectomy, salpingectomy). This “surgical control” group is included to eliminate the possibility that surgical interventions in the pelvic region affect levels of cardiovascular disease markers over time.
4. *Pre-menopausal women, no surgery.* It is expected that women in this non-surgical, pre-menopausal control group will not exhibit remarkable changes in cardiovascular disease markers during the study’s time frame, and as such, will be used to compare with both groups of pre-menopausal women having undergone surgery (PSO or other pelvic surgery).

Patients with pre-existing malignancy or who have experienced a myocardial infarction event in the year prior to their surgery will be excluded from the study since these populations exhibit elevated HSP27 serum levels [63, 87–90]. Once patients decide to enroll in the study and provide signed informed consent, they will undergo a standardized cardiac consultation that includes a detailed personal health history (e.g., medication use, incidence of hypertension, diabetes mellitus, and prior cardiovascular events), cardiovascular physical examination and an ECG. Patients will then be asked to give blood and urine samples before and at various times after surgery (University of Calgary). Retinal images will also be captured before and after surgery (Calgary Retina Consultants). The time commitment required for participation in this study is 1 year (following surgery), with an option to extend participation for an additional year.

### Participant recruitment

Depending on family history and outcome of genetic testing, women that are positive for mutations in BRCA1/2 or Lynch syndrome associated-genes (e.g., HNPCC) may be referred by the University of Calgary’s team of genetic counselors for an information sharing consultation with gynecologists who specialize in minimally invasive PSO. During the medical genetics consultation, patients will be given a cursory introduction to this study via dissemination of an information pamphlet. After discussing with their gynecologist, who will further elaborate on the details of this study, patients who decide to undergo PSO and are interested in participating will then be scheduled for an appointment with a cardiologist (EOB). Post-menopausal women considering PSO and pre-menopausal women undergoing other pelvic surgeries will be introduced to the study by the same team of medical genetics counsellors and gynecologists, while non-surgical control participants will be enrolled through the use of recruitment flyers posted at the University of Calgary and its affiliated hospitals. Active patient enrollment for this study will take place over 3 years. Recruitment targets for each of the four patient treatment groups are 20 per year, totalling 240 participants for the duration of the study.

### Data collection

Cardiac consultations will take place before and at 6- and 12-months after surgery, during which patients will be given an ECG and monitored for incident hypertension, diabetes mellitus, and cardiovascular events (e.g., myocardial infarction, stroke). Fasting blood and urine samples will be collected before surgery (PSO or other pelvic) and at 1, 3, 6, and 12 months after surgery. In addition to routine blood tests (glucose, hemoglobin A1c), Calgary Lab Services will conduct the following analyses: lipid profile (total cholesterol, HDL, LDL, triglycerides) and a female endocrine panel (estradiol, progesterone, FSH, LH). Our research laboratory will separately analyze blood samples at these time points for assessment of novel cardiovascular biomarkers. Plasma will be extracted from blood samples, aliquoted, and stored at -80 °C for future testing (e.g., enzyme-linked immunosorbent assays for HSP27, HSP27 antibodies, PCSK9, as well as inflammatory cytokine arrays [performed by EVE Technologies on campus]). Urine samples will also be stored at -80 °C for future isolation of microparticles and exosomes (ultracentrifugation) and detection of HSP27 and PCSK9 (Western blot and mass spectrometry). Retinal images will be captured by digital photography before and at 6 and 12 months after surgery by a high-volume retinal referral clinic (Calgary Retina Consultants) and images will be securely transferred to the University of Wisconsin Retina Core lab for analysis (Drs. Ron and Barbara Klein). In addition to the above data collection time points, patients will be given the option of extending their participation for an additional year, which will involve cardiac consultations, blood draws, and retinal imaging at 18- and 24-months post-surgery.

To complement this work, our collaborator, Dr. Denise Nebgen (MD Anderson, Houston; **W**omen choos**I**ng **S**urgical **P**revention “**WISP**” trial [ClinicalTrials.gov Identifier: NCT02760849] researcher) will also provide plasma samples before and after PSO in their cohorts diagnosed with BRCA- or Lynch syndrome-associated mutations (~ 35 WISP participants).

### Outcomes

The primary outcome is a change in levels of HSP27 following PSO. Plasma HSP27 will be measured using ELISA methods before and 1, 3, 6, and 12 months following surgery. Since previous findings have shown that higher HSP27 serum levels correlate with cardiovascular health [66], it is likely that women experiencing premature menopause after PSO will experience a worsening of their cardiovascular health (as measured by total cholesterol and LDL) and consequently, lower levels of HSP27. HSP27 localized to microparticles isolated from plasma and urine will also be measured using Western blotting and mass spectrometry. Additional primary outcomes assessed at the same time points and analyzed by ELISA are: HSP27 natural antibodies, which appear to be elevated in healthy controls compared to coronary artery disease patients, and PCSK9, which itself may be regulated by HSP27 (unpublished data). Secondary outcome variables are lipids (total cholesterol, triglycerides, HDL, LDL), ovarian hormones (estradiol, FSH, LH, progesterone), cytokines, and retinal vessel (arteriole and venule) diameters. Abnormally high lipid levels and/or severe post-menopausal symptoms following PSO may necessitate alterations in management and treatment by attending physicians.

### Sample size calculation

To date, there are no prospective studies investigating HSP27, HSP27 Ab, and PCSK9 levels in women following PSO and the ensuing premature, surgical menopause. To estimate the necessary sample size, we assumed an effect size of 0.3, Type 1 error rate α = 0.05, and desired power = 0.90. Based on our conservative assumptions, a minimal sample size of at least 160 participants (40 per group) is needed for the study. Assuming 10% attrition rate over the course of the study, a minimum of 178 participants will be required (which is well below our recruitment target of 240). Once there is sufficient enrollment (*n* = 6 / group), effect size will be recalculated based on pilot data (again presuming α = 0.05 and power = 0.90) and the sample size adjusted accordingly as the study continues.

### Statistical analyses

Data analysis will be performed using Statistical Analysis System (SAS) software. Probability (p) of less than 0.05 will be considered significant. Experimental outcomes for continuous variables will be expressed as means ± standard deviation for each time point and the variance between the groups will be analyzed using ANOVA. Categorical variables will be compared with a chi-square test. In order to examine the relationship between variables, regression analysis will be performed. If more than one predictor variable is found to affect the outcome, multivariate regression will be employed to identify significant associations.

### Patient engagement activities

**Fig. 2:**
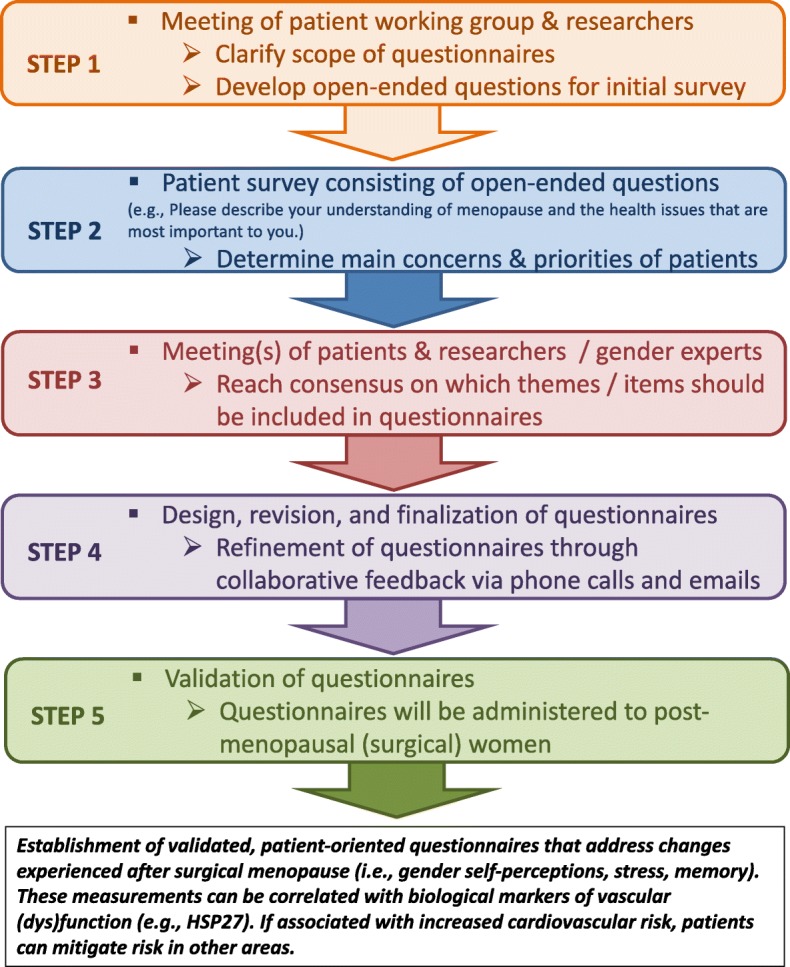
Workflow of development of questionnaires that reflect patient priorities

In addition to identifying biological markers associated with cardiovascular dysfunction in women experiencing surgical menopause, we are also interested in understanding how shifts in gender perception (which includes subjective measurements of stressful experiences) can heighten CAD risk. The idea that gender (a construct distinct from “sex” that encompasses social perceptions and expectations regarding the roles of men and women) can shape the clinical outcome of CVD patients is illustrated by a recent study using the GENESYS Gender Index [91, 92], suggesting that a feminine gender score, but not female sex, was associated with heart disease risk factors. We thus hope to identify how surgical menopause influences the subjective health perceptions, feelings, and lifestyle changes of women after PSO, and if these can affect their vascular health. Together with a focus group of women in Calgary that have undergone PSO, we will co-create questionnaires exploring changes in perceptions of the patient experience (Fig. 2). We will also plan a research forum open to the general public that will address the health concerns most relevant to PSO patients.

### Study limitations

We recognize the importance of incorporating a Quality of Life (QOL) assessment that collects data on diet, exercise, and other lifestyle measures. Although there is no formal component in the pilot phase of this study, we plan to include this in the future. Additionally, one of the patient engagement working group’s goals is to generate questionnaires together with our research team that directly reflect the patient experience following PSO – these will contain questions on self-reports of lifestyle changes. We also acknowledge that sample size in this study is small; however, it can provide the basis for future, larger studies involving multiple centres.

## Discussion

Women with hereditary cancer mutations increasingly elect to undergo PSO as a risk-reducing treatment option, but it is important to be aware of potential ensuing cardiovascular health consequences. As the association of cancer risk with a particular mutation is not fully characterized, the advantages and disadvantages of PSO must be carefully weighed. Besides increased risks of cardiovascular disease and cognitive dysfunction, there are also other associated morbidities that are elevated after PSO, and adjustments to changes in lifestyle (e.g., sexual performance [93]) and in gender self-perceptions are additional issues that should not be ignored.

**Fig. 3:**
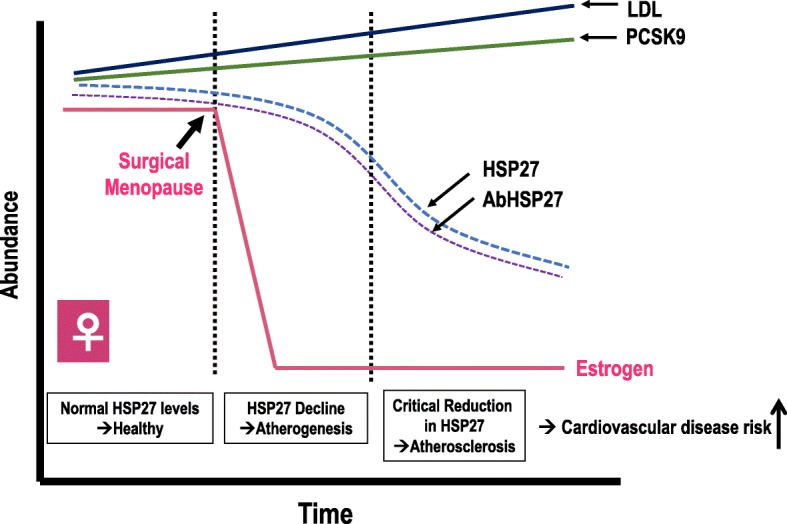
Proposed interplay of HSP27 & estrogens

The “biological arm” of this study will determine how the abrupt loss of estrogen after PSO influences levels of established (e.g., lipids) and novel biomarkers (e.g., HSP27, retinal vessel diameters) in pre-menopausal women. We predict that after surgery, the sudden drop in estrogen will lead to gradual increases in LDL and PCSK9, with concomitant decreases in HSP27 and HSP27 Ab, making these women more susceptible to cardiovascular disease risk (Fig. 3). Results from this pilot work will inform future studies involving larger patient populations, with the long-term goal of using these markers as predictors of cardiovascular health in susceptible populations. Ongoing in vivo studies in our laboratory using an atheroprone murine model suggest that ovariectomy leads to an exacerbation of atherogenesis and that HSP25 (the murine orthologue of HSP27) can substantially reduce the development of aortic atherosclerosis. Hence, HSP27 may serve not only as a marker for cardiovascular disease but possibly as a novel therapeutic in post-menopausal women.

## Abbreviations

BRCA1/2: Breast cancer susceptibility gene 1/2
CAD: Coronary artery disease
CVD: Cardiovascular disease
HSP27 Ab: HSP27 antibodies
HSP27: Heat shock protein 27
MHT: Post-menopausal hormone therapy
NO: Nitric oxide
PCSK9: Proprotein convertase subtilisin/kexin type 9
PSO: Prophylactic salpingo-oophorectomy
WISP: Women choosing surgical prevention trial
